# Aotearoa genomic data repository: An āhuru mōwai for taonga species sequencing data

**DOI:** 10.1111/1755-0998.13866

**Published:** 2023-09-15

**Authors:** Ben Te Aika, Libby Liggins, Claire Rye, E. Owen Perkins, Jun Huh, Rudiger Brauning, Tracey Godfery, Michael A. Black

**Affiliations:** ^1^ Research and Enterprise Office University of Otago, Dunedin New Zealand; ^2^ School of Natural Sciences, Massey University Auckland New Zealand; ^3^ Genomics Aotearoa New Zealand; ^4^ New Zealand eScience Infrastructure University of Auckland Auckland New Zealand; ^5^ Invermay Agricultural Centre, AgResearch Ltd, Mosgiel Otago New Zealand; ^6^ Department of Biochemistry University of Otago Dunedin New Zealand

**Keywords:** benefit‐sharing, CARE principles, data repository, indigenous data sovereignty, Māori

## Abstract

The Aotearoa Genomic Data Repository (AGDR) is an initiative to provide a secure within‐nation option for the storage, management and sharing of non‐human genomic data generated from biological and environmental samples originating in Aotearoa New Zealand. This resource has been developed to follow the principles of Māori Data Sovereignty, and to enable the right of kaitiakitanga (guardianship), so that iwi, hapū and whānau (tribes, kinship groups and families) can effectively exercise their responsibilities as guardians over biological entities that they regard as taonga (precious or treasured). While the repository is designed to facilitate the sharing of data—making it findable by researchers and interoperable with data held in other genomic repositories—the decision‐making process regarding who can access the data is entirely in the hands of those holding kaitiakitanga over each data set. No data are made available to the requesting researcher until the request has been approved, and the conditions for access (which can vary by data set) have been agreed to. Here we describe the development of the AGDR, from both a cultural perspective, and a technical one, and outline the processes that underpin its operation.

## 1 INTRODUCTION

Te ao Māori, the worldview of the Indigenous people of Aotearoa, New Zealand, recognizes the interconnectedness of all things, both living and non‐living (Our Land and Water National Science Challenge, 2022). There are some key philosophies that help explain this connectedness. First whakapapa, often described as the genealogical connection between people and as creating an intergenerational hereditary responsibility for both past and future generations. Whakapapa establishes genealogy, social and ecological relationships, cultural histories, family traits and ancestral inheritances (Hudson et al., 2016). The ecological relationships are key, how an individual, a whānau, hapū and iwi (family, clan and inter‐clan groups respectively) are placed through whakapapa within the greater ecosystem: the land and water, the fauna and flora (Ministry for the Environment & Stats NZ, 2021). A second philosophy is taonga, translating to treasure, anything prized, including socially and culturally valuable objects, resources, phenomena, ideas and techniques (Māori Dictionary Project, 2022). The notion of taonga is key to understanding the significance of the environment to Māori. All living species are considered taonga and therefore require protection through kaitiakitanga (guardianship; Hudson et al., 2016), with this responsibility extending to any data generated through the study of taonga species. For genomic data derived from taonga species, there is also the potential to obtain additional information about whakapapa, which is itself taonga, and must also be protected as part of the kaitiakitanga role. A key component of this role, therefore, is the ability to maintain control over such data. The creation of appropriate locally based data storage facilities offers crucial protection for Indigenous communities and enables any data sharing to occur safely and with respect for Indigenous sovereignty.

The sharing of scientific data is a key feature of the Western science system, and in many disciplines there are strict requirements (from funding agencies, institutions, data repositories, and journals) around ensuring the availability of data associated with specific research outputs (Fecher et al., 2015). This is certainly true in fields where high‐throughput sequencing technologies are heavily utilized, with many journals now strictly mandating the deposition of unprocessed sequence data into large‐scale data repositories (e.g. encouraged by the Joint Data Archiving Policy (Dryad, 2022)). In most studies involving non‐human subjects, access to these data sets is unrestricted, with access provided through simple web‐based interfaces (e.g. the International Nucleotide Sequence Database Collaboration, (Cochrane et al., 2016)) without any attempt to control who downloads the data, or what the data may subsequently be used for.

Such an approach sits comfortably within the framework of Open Science, which strives to ensure the reproducibility of scientific findings by making all aspects of the research process open and accessible to all researchers, including data (e.g. the FAIR Guiding Principles for scientific data management and stewardship, (Wilkinson et al., 2016)). This level of openness, however, is very much a feature of a Western‐influenced scientific system, and in many cases—particularly those involving genomic data derived from samples of indigenous flora and fauna—directly conflicts with the traditional beliefs and values of the Indigenous peoples for whom these species hold a strong cultural importance (Hudson et al., 2020).

Historically, this Western science approach to research has dominated, with the rights and beliefs of Indigenous peoples either ignored or marginalized, in exchange for the perceived greater good that openness and sharing would more rapidly lead to further scientific advancement and collective benefit. Recently, however, there has been increasing dialogue occurring within the international scientific community about the ways in which Indigenous rights and values can be incorporated into the scientific process, and how Indigenous researchers should be in leadership roles in projects that relate to species of cultural significance—this might increase the likelihood of Indigenous rights and values being properly incorporated into the research, and that Indigenous standards are being upheld (Golan et al., 2022; Liggins, Hudson, & Anderson, 2021; McCartney et al., 2022). It is important to note, however, that not all Indigenous researchers are cognisant of, or sympathetic to, Indigenous values (a disconnect that in many cases will be a legacy of colonization), so this should be treated with some caution—cultural expertise is the crucial factor here.

In Aotearoa New Zealand, this dialogue has been evolving slowly, over many years. The existence of Te Tiriti o Waitangi (The Treaty of Waitangi) as our nation's founding document has (albeit gradually) influenced policy decisions made by the New Zealand Government, to the extent that concepts such as “Māori consultation” and Vision Mātauranga, have been firmly embedded within the research landscape of Aotearoa for many years. This movement towards increased involvement of Māori in all aspects of research has accelerated rapidly over the past 2–3 years, with multiple frameworks now available that provide guidance on how to undertake research in partnership with Māori (e.g. Cunningham, 2000; Hudson et al., 2020, 2016, 2021), although it is unclear how frequently such frameworks are used by researchers. It is with this view, through a “Māori lens”, that we need to consider the implications of using, storing and sharing genomic data derived from taonga species and this requires different approaches to blanket decisions on open data (Hudson et al., 2020; McCartney et al., 2022).

For scientists involved in molecular ecology research in New Zealand, the study of indigenous organisms has long required consultation with those whānau, hapū and iwi who hold kaitiakitanga over the taonga species found within their rohe (region). Ideally, this consultation process would be a true partnership, where scientists and mana whenua (Indigenous territorial authority holders) would work side by side, conducting important research that would ultimately benefit both Māori communities (e.g. improved conservation outcomes for an endangered species), and the scientific community as a whole. In practice however, consultation has sometimes been seen as an exercise in “permissioning” and once permission has been obtained, Western science proceeds with “business as usual”. In these situations, there is often little feedback returned to the Māori communities with whom consultation was undertaken, and the primary benefits from the research—scholarship, recognition, citation—are vested only in the researchers who publish the work.

In this context, the generation of genomic data from taonga species creates an additional level of complexity, particularly when considered within the framework of te ao Māori (Collier‐Robinson et al., 2019). Until recently, this disconnect between traditional belief systems and the “open data” requirements associated with scientific publications (not to mention the persistence of the “publish or perish” mentality that still pervades the Western science system) meant that many researchers were faced with a difficult choice. They could either make genomic data freely available via an offshore open‐access repository (and effectively negate the kaitiakitanga status guaranteed by Te Tiriti), or limit the impact of their research by maintaining data sovereignty, and publishing in a journal that may not be as highly regarded in their field. In addition, with recent technological developments, the latter choice has become even more difficult—one of the attractions of submitting data to a centralized open repository is that it then removes the burden of long‐term data preservation from the researcher—with raw data from a relatively small‐scale sequencing study now able to consume multiple terabytes of compressed storage, just funding the ongoing non‐public archival storage of genomic data can rapidly become problematic for both researchers and their host institutions.

It is before this backdrop that a national‐scale resource for the storage of genomic data from taonga species has been developed. The Aotearoa Genomic Data Repository (AGDR) is part of a government‐funded initiative, Genomics Aotearoa (GA), which aims to develop the infrastructure needed to underpin future genomic research in New Zealand. This work was undertaken in partnership with another national initiative, the New Zealand eScience Infrastructure (NeSI), which provides national‐level compute, storage and technical expertise to support the New Zealand Science system. Together GA and NeSI have worked to build a prototype resource which provides a secure, NZ‐based option for the storage, management and sharing of non‐human genomic data generated from biological and environmental samples originating in Aotearoa New Zealand. Here, we provide an overview of the approach and development activities in support of the Aotearoa Genomic Data Repository, and describe the benefits of the repository, along with proposed future developments . Throughout the text, a number of Māori terms and phrases are used. Box 1 contains a brief explanation for each of these.

BOX 1 **Key words and phrases.**

**Table:** 

Āhuru mōwai	Calm place, safe haven.
Aotearoa	Land of the Long White Cloud. One of the original names for the islands that became known as “New Zealand” after European arrival.
Hapū	Indigenous autonomous clan or kinship group.
Iwi	Amalgamated kin‐based grouping (e.g. an alliance of related Hapū).
Kāhui Māori	Oversight group.
Kaitiaki/Kaitiakitanga	Guardian/guardianship.
Mana whenua	Authority held by tangata whenua in a particular rohe.
Mana	Prestige, authority, control, power, influence, status (in‐depth descriptions of “mana rangatira” and “mana kaitiaki” are provided in the section: Principles underlying the cultural workstream).
Manaaki	To support, take care of, and uphold the mana of others.
Māori	A collective term for the Indigenous people of Aotearoa New Zealand.
Mātauranga	Traditional knowledge system, ways of knowing, values and customs, methods and practices associated with knowing.
Rangatira	To be of high rank, esteemed, revered, of chiefly status. Leader and authority holder.
Rohe	Geographic region.
Tangata whenua	Indigenous people of the land—casual term, poetic phrase for territorial kin group or individual.
Taonga	Valued, treasures, treasured things.
Te Tiriti o Waitangi	The Treaty of Waitangi.
Te ao Māori	Māori world view. The ‘organizing principle’ or ‘values lens’ through which the world is coloured, viewed, observed, understood and reality determined.
Tikanga	Māori traditional values, protocols and practices. Māori law.
Vision Mātauranga	A policy framework developed by the NZ Government which provides strategic direction for research that is of relevance to Māori. It aims to give effect to the Treaty of Waitangi by enabling better incorporation of Māori knowledge, resources and people into Government‐funded scientific research.
Whakapapa	Ancestry, genealogy
Whānau	Family
Whare Wānanga	Traditional Māori institutions of higher learning

## 2 BUILDING THE AGDR

The main goal in establishing the Aotearoa Genomic Data Repository was to provide a resource that enabled both researchers and kaitiaki (i.e. those individuals or groups with a guardianship role) to fulfil their responsibilities regarding genomic data sets in which they have been involved in generating. Although motivated by and focused on the Aotearoa New Zealand context, the AGDR supports the globally relevant FAIR Principles (Wilkinson et al., 2016) and the CARE Principles (Carroll et al., 2021). For researchers, this involves providing a mechanism by which data can be stored, managed and shared, while also meeting the accessibility and availability requirements specified by the journals in which the research is to be published. For those with kaitiaki roles, these researcher‐centric capabilities needed to be balanced against the responsibilities associated with kaitiakitanga, and with the principles of Indigenous Data Sovereignty, which largely follow from the minimum standards adopted in the United Nations Declaration on the Rights of Indigenous Peoples (United Nations General Assembly, 2007). The overarching concept that has guided this work can be clearly encapsulated by the phrase “Māori control of Māori data” (see Box 2).

BOX 2 **Māori data sovereignty.**

The topic of Indigenous Data Sovereignty has received increasing attention and discussion in the scientific community in recent years, with growing acceptance that Indigenous peoples have a right to exercise control over their own data, including the results and narratives derived from its use. This is reflected perhaps most notably in the development of the CARE Principles for Indigenous Data Governance (Carroll et al., 2021), which provide principles to guide the management of data in a way that protects the rights and interests of Indigenous Peoples. In Aotearoa New Zealand, Te Mana Raraunga (the Māori Data Sovereignty Network) defines Māori Data Sovereignty as “the inherent rights and interests that Māori have in relation to the collection, ownership, and application of Māori data”, and defines Māori Data Governance as referring to “the principles, structures, accountability mechanisms, legal instruments and policies through which Māori exercise control over Māori data” (https://www.temanararaunga.maori.nz). These rights and interests are held and exercised by Te Tiriti authorities—a concrete example in the context of the AGDR is the kaitiaki role held by Rangitāne o Manawatū in relation to the Mānuka data set (see Box 6), with the principles of Māori Data Sovereignty upheld through the AGDR's kaitiaki‐based data access protocols. An example illustrating how the AGDR has helped to support an existing kaitiaki partnership is described in Box 7.

Although not published when the development of the AGDR began, the document “Te Nohonga Kaitiaki ‐ Guidelines for Genomic Research on Taonga Species” (Hudson et al., 2021) emphasizes the importance of Māori Data Sovereignty in the context of genomic research, as does the document “A WAI 262 Best Practice Guide for Science Partnerships with Kaitiaki for Research Involving Taonga” (Potter, 2022), which more broadly addresses the role of kaitiaki and scientists in research partnerships involving taonga, providing a solid framework for researchers engaging with mana whenua in this space.

Three areas of data sovereignty are notable from the sources above:

1. National data sovereignty—data which are important to the nation state
2. Indigenous data sovereignty—data which are important to Indigenous peoples
3. Globalized data—data which have been made openly available and sits outside of national bounds, therefore the enforcing (or honouring) of Te Tiriti is practically limited

Sovereignty is important to understand as a basis for working with applied data management systems within an Indigenous setting.

From an Indigenous perspective data sovereignty relates to control of data, or the ability to make decisions regarding access and use of any data that is important to the respective Indigenous community. Who makes these decisions is established through whakapapa descent. As Indigenous people Māori occupy diverse geographic areas and are not geo‐politically aligned, a localized lens and the ability to identify iwi or hapū who hold mana whenua status over the research area or taonga species concerned is therefore imperative when determining decision‐making authority.

In order to develop these linked but potentially conflicting capabilities, two distinct workstreams were established, one cultural, led by the Genomics Aotearoa Vision Mātauranga (VM) Coordinator, and one technical, comprising a mixed team of NeSI and GA project members, chaired not only by the head of the GA Bioinformatics leadership team but also including the VM Coordinator to provide a Māori perspective on the development work being undertaken.

### 2.1 Principles underlying the cultural workstream

The primary goal for the work undertaken in this component of the project was the development of culturally appropriate protocols for the operation of the data repository, with respect to both data submission requests, and data access requests. In developing these protocols, a key goal was to capture the significance and importance of Mātauranga Māori, Mana Rangatira and Mana Kaitiaki. These concepts, including how they relate to each other, are discussed in the following paragraphs.

Mātauranga Māori describes the Indigenous system and body of knowledge which originates from the ancestors, including the ways of knowing, the Māori world view and perspectives, as well as Māori creativity and cultural practices (Māori Dictionary Project, 2022). Mātauranga Māori has developed out of the need to describe the Indigenous ‘ways of knowing’ in comparison to Western science, evolving through many generations and centuries of both accumulated lived experience and formalized institutional learning. This sphere of knowledge evolution and development evolved entirely without European influence, until the arrival of the HMS Endeavour, a Royal Society science mission in 1769. At that time, and subsequently until the arrival of the Doctrine of Discovery and then colonization, all knowledge and knowledge systems within Aotearoa sat under the authority, or mana, of the various Indigenous societal institutions in Aotearoa; the mana of those societal institutions was not surrendered or ceded through the signing of Te Tiriti o Waitangi. This realization sets in place the constitutional underpinnings of authority in relation to education, science research and mātauranga.

Mana in relation to research, data and information remains a key focal point for Māori interests. For many centuries, knowledge within Aotearoa sat exclusively within the sovereign authority of the Indigenous peoples and their socio‐political systems. Māori knowledge systems included the Whare Wānanga—traditional graduated houses of learning. Mātauranga Māori comes with its own set of rules, or tikanga, which need to be observed during the experience of learning. These tikanga have been re‐contextualized by Indigenous scholars within contemporary settings in response to the colonizing influence of Western science (Jackson, 2019). Jackson articulated that the concept of mana provided a constitutional‐type basis for political power as an absolute authority (akin to sovereignty) which is the prerogative of Indigenous institutions. He describes these attributes of mana or powers as:

a power to protect – that is the power to protect, manaaki and be the kaitiaki for everything and everyone within the polity; a power to define – that is the power to define what should be protected, and the power to define the rights, interest and place of individuals and collectives; a power to decide – that is the power to make decisions about everything effecting the wellbeing of the people; and a power to develop – that is the power to change to meet new circumstances in ways that are consistent with tikanga and conducive to the advancement of the people. ‐ Moana Jackson, 2019

The matter of mana is rightly considered the constitutional level basis for tikanga or correct Indigenous knowledge practices associated with research and data use. Mana mātauranga, or the power in relation to Indigenous knowledge, information and ways of knowing, could, therefore, be described as: the power to decide what is worth investigating, why, how and by whom it should be investigated, and with whom to share knowledge with.

Rangatiratanga, as an authority to exercise mana or power, includes a relationship element of care. For kaitiaki, this care or manifestation of kaitiakitanga, can only be effective when there exists the authority or mana to care through acting as a decision‐maker. Māori giving advice to non‐Māori decision‐makers cannot be considered Māori decision‐making, and thus has a very limited basis for meeting a threshold where the decision‐making honours Te Tiriti and supports the Māori–Crown relationship. Kaitiakitanga has been adopted into some key elements of legislation and administrative doctrine such as the Resource Management Act 1991 and the Conservation Act 1987, however, it is debatable whether these legal manifestations of kaitiakitanga effectively support the traditional role of mana kaitiaki or honour Te Tiriti and support the Māori–Crown relationship. Given their legislative significance, it is important to examine the traditional use of these terms. Alongside the attributes of power already provided by Jackson (2019), the power to protect, or the mana of kaitiaki, is tightly bound to mana rangatira, the power to make decisions. We note the following identified authorities:

Mana rangatira: the power and authority to decide, which also includes:

Mana kaitiaki: the power to protect and decide what is protected regarding all things affecting Māori interests and Māori wellbeing.

**FIGURE 1:**
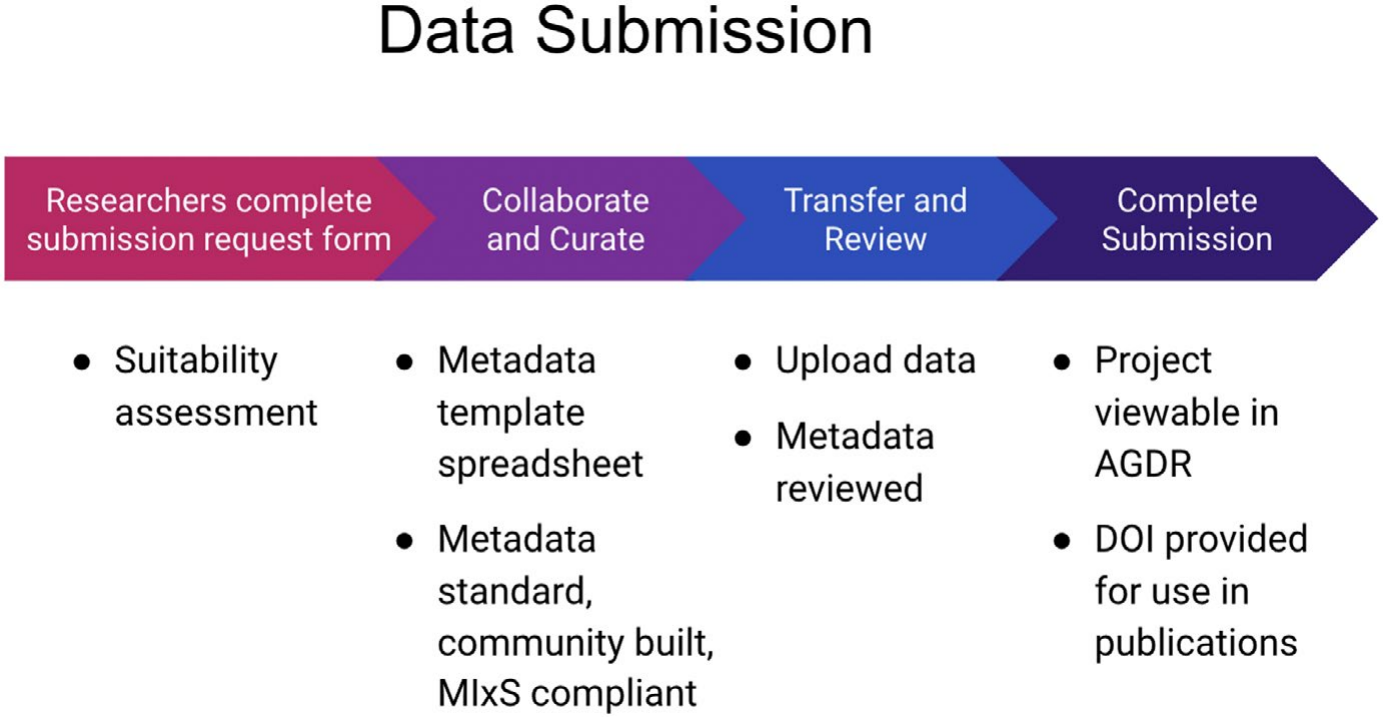
Process for submitting data to the Aotearoa Genomic Data Repository (AGDR). Individual data sets are assessed for their suitability for the AGDR based on information submitted by the researcher (Box 3), including what species they are derived from. Researchers are required to provide metadata that meets community standards (e.g. complying with the Genomic Standards Consortium Minimum Information about any (x) Sequence, MIxS) with their genomic sequences. Following data upload and metadata review by the AGDR team, the researchers are provided the opportunity to create a DOI for their data and to view their project in AGDR.

These traditional notions are important components of the information that is collected when data sets are submitted to the AGDR. Both the specifics of processes followed for Māori consultation and the contact details for kaitiaki representatives are requested from data submitters. This information is also used when requests for data set access are received. Figure 1 outlines the process for submitting data to the AGDR, while Box 3 contains the list of information that is collected from submitters.

BOX 3 **Information requested as part of the data submission process.**

*Submitter details*:

1. Email

*Project Details*:

2 Project Title
3 Description
4 Approximate size of the data set
5 Date collected
6 Investigator name
7 Contact Info
8 Affiliation
9 Support Source (optional)

*Data sovereignty and Kāhui Māori considerations*:

10 Species name
11 Sample area name
12 Sample site GPS
13 Māori written consent received
14 Māori Kaitiaki group name
15 Māori Kaitiaki representative name
16 Māori Kaitiaki representative email
17 Māori Kaitiaki phone number
18 Research institution officer name
19 Research Institution officer contact email
20 Other details (Please provide us with any other details that may help us.)

**FIGURE 2:**
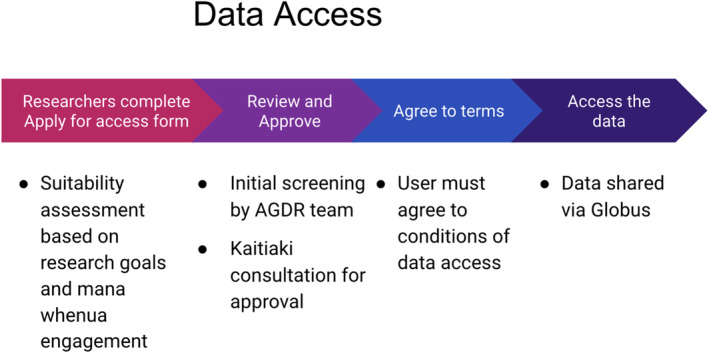
Process for requesting data from the AGDR. Requesting researchers are required to answer a series of questions (see Box 4) that help the AGDR team to screen the request for complete information and for kaitiaki to make a decision regarding whether access should be approved. The requesting researcher will need to agree to the conditions of access (see Box 5) prior to being able to access the data.

The processes for making data access requests vests all decision‐making authority with those holding mana rangatira, or their mandated proxy. In order to inform this decision‐making process, researchers who are requesting access to a specific data set are required to answer a series of questions as part of their access request. The process is outlined in Figure 2, with the specific questions presented in Box 4. These were designed to help kaitiaki gauge the specific risks and benefits associated with providing access to a specific data set (e.g. benefits may involve: improved knowledge to aid with species conservation, access to new methods for environmental monitoring, financial gain from a joint commercial venture; whereas risks could involve: loss of control of data, reputational damage or even exploitation through biopiracy).

BOX 4 **Information requested as part of the access request process.**

1. Email address.
2. Name of data set for which access is being requested.
3. Does the application include, or is it covered by, a written consent from the Originator or delegated Māori authority holder? Please provide any related comments or information that will help us evaluate this component.
4. Have you undertaken consultation or engagement with Māori about the research you wish to undertake with this data set? Please provide any related comments or information that will help us evaluate the suitability of any consultation that has been undertaken.
5. Describe how risks to Māori relationships, values, rights and interests will be addressed in the research that you are proposing.
6. Are Recombinant DNA techniques involved in the research that you are proposing? If you answered Yes, please provide details.
7. Describe the benefits to the Originator, whanau and/or hapū most directly affected by the research. If there are no benefits for Māori associated with your research, please state this.
8. Is your research likely to result in any of the following: ownership claims, development of new intellectual property, involvement of investment partners and/or funders/co‐funders? If you answered Yes, please provide details of how these issues will be resolved, and in particular how they will impact and/or benefit Māori.
9. Please describe any areas of cultural and/or commercial sensitivity that are associated with your proposed project.
10. Does your project align with the principles outlined in Te Mata Ira: Guidelines for Genomic Research with Māori? Please provide additional relevant information to support your answer.
11. Full name / Institution / Project Title.
12. Project summary (including reasons for needing access to this data set).
13. Estimated start and end dates.
14. Whether funding has been secured to support the project. If Yes, details of funding sources.
15. Project team members (full name, affiliation, and email addresses), and collaborating researchers.
16. Whether associated phenotypic data are required.

Once the information in Box 4 has been collected, this is passed on to the kaitiaki contact for the data set in question. If additional information or clarification is required as part of the decision‐making process, this can be requested from the researcher (i.e. the requestor) through the AGDR (i.e. the kaitiaki do not have to interact directly with the researcher, although they do have this option if that is their preference). If a researcher's access request is approved by the kaitiaki for that data set, the approval information is recorded by the AGDR team, and the researcher is then asked to agree to the data usage conditions associated with that data set. These conditions can be specific to each data set, or even to each access request; it is at the discretion of the kaitiaki to define any usage restrictions of additional conditions. By default, the conditions are relatively non‐permissive, and are geared towards non‐commercial research applications. An example of a typical set of conditions is presented in Box 5.

BOX 5 **Example of usage conditions associated with data set access.**

Condition 1: Kaitiaki Māori and Aotearoa Genomic Data Repository need to be informed of any changes to the research programme, prior to those changes being made.

Condition 2. You will need to ensure that the commitments made in the application are met. Particularly that the outcomes for Māori are highlighted in publications and reported to the Aotearoa Genomic Data Repository and Kaitiaki Māori.

Condition 3. You will ensure that there will be no sharing of data with any other party, without prior informed approval of the Aotearoa Genomic Data Repository and Kaitiaki Māori. This includes depositing the data in any online repository or archive, which is specifically prohibited.

Condition 4. Any commercial opportunities which arise or are discovered will be brought to the attention of the Kaitiaki Māori prior to any announcement or disclosure to any other party. You will offer the Kaitiaki Māori the opportunity to be a beneficiary or business partner in any commercial opportunity.

Condition 5. Access to the data is granted for a 12 month period, expiring on XXX. If you still require access to the data after this date, please contact the Aotearoa Genomic Data Repository prior to YYY to arrange for an extension. Once your access to the data set has expired, you are required to delete all copies of the data set, and provide the Aotearoa Genomic Data Repository with a brief description of the work that was undertaken with the data set.

Condition 6. Any publications relating to your use of these data need to include acknowledgement of Kaitiaki Māori, the Aotearoa Genomic Data Repository, Genomics Aotearoa, and the New Zealand eScience Infrastructure for facilitating access to this data set. Please send details of these publications to the Aotearoa Genomic Data Repository contact email address.

BOX 6 **Case study: Mānuka genome assembly.**

Mānuka (*Leptospermum scoparium*) is a flowering plant from the Myrtaceae family which grows throughout Aotearoa. It is considered taonga by Māori, and its nectar is used in the production of Mānuka honey. In 2019, scientists from the Plant and Food Research (a Crown Research Institute funded by the New Zealand Government) reported the first assembly of the Mānuka genome (Thrimawithana et al., 2019), using samples obtained from the rohe of Rangitāne o Manawatū. As described by Morgan et al. (2019), engagement with mana whenua occurred “after the fact”, but this (albeit belated) consultation process led to a consensus view that the genomic sequence of Mānuka should not be made freely available, despite this being the typical approach within the Western science paradigm. Instead, the Mānuka genome assembly is hosted by the AGDR, and access can be requested from the Mānuka project page within the repository. These requests are passed to a kaitiaki contact for Rangitāne, who returns access decisions once the merits of each request have been considered.

BOX 7 **Case study: Sequencing Kākāpō.**

The Kākāpō (*Strigops habroptilus*) is a flightless ground‐dwelling parrot that is endemic to New Zealand, and is taonga to Māori. The species is critically endangered, with only 252 birds currently alive, and the New Zealand Department of Conservation (DOC), along with many other organizations and individuals, plays a critical role in the Kākāpō Recovery Programme, working to improve both the size and the overall health of the kākāpō population. As part of this initiative, DOC has undertaken extensive DNA and RNA sequencing of kākāpō, with full genome sequence available for a substantial proportion of the population. These data are stored within the AGDR, however the access mechanism is through interaction with the Department of Conservation. When a researcher clicks on the “Apply for access” button on the AGDR kākāpō project page, they are forwarded to the data access request page on the DOC website. The application process is fully managed by the DOC in partnership with Te Rūnanga o Ngāi Tahu, the kaitiaki for this data set. If access is approved, this information is communicated back to the AGDR team, which then facilitates access for the approved researcher(s).

### 2.2 Principles underlying the technical workstream

The primary goal for the work undertaken in this component of the project was to capture the technical requirements for a national genomic repository, then identify and deploy an appropriate system on an existing national compute and storage infrastructure. Mātauranga Māori and Mana Kaitiaki were considered closely when making decisions. Furthermore, the mana attributes—power to protect, power to define, power to decide and power to develop—had major implications on the technological solution that was employed. For example, one of the key decisions was the location of storing the sequence data. We determined it was most appropriate to store it locally in Aotearoa New Zealand in order for mana whenua to retain tangible physical and legislative power over the data.

In terms of approaches to building AGDR, we could build a bespoke platform, or we could utilize existing open‐source technologies in this space. As an initial step, a review of suitable technology options was carried out with the following repositories examined:

- Genomics England Airlock: https://research‐help.genomicsengland.co.uk/display/GERE/5.+The+Airlock
- EGA archives: https://ega‐archive.org/
- National Cancer Institute (NCI) genomic data commons: https://portal.gdc.cancer.gov/
- Bioplatforms Australia OZ Mammals Genomics: https://data.bioplatforms.com/organization/bpa‐omg
- Repositories listed on the Elixir directory: https://elixir‐europe.org/platforms/data/core‐data‐resources

From here the options were narrowed down to either Gen3 (https://gen3.org/), or CKAN (https://ckan.org/). Gen3 is an open‐source genomic data repository solution developed at University of Chicago in collaboration with NCI for their Genomic Data Commons, while CKAN is a more general open‐source data management system, developed with Government and Enterprise scale in mind, as utilized by Bioplatforms Australia and many others.

CKAN had the advantage of being more widely used and having a better administration interface, while Gen3 had much more domain specificity and a strong developer community. Given the development resources available and two suitable technology choices available, it was decided that utilizing one of these, rather than building from scratch, was the sensible choice.

NeSI, as an organization with expertise in providing technical infrastructure, concluded that there would be more benefit from a solution that focuses on the genomics and bioinformatics domains, thus resulting in the Gen3 system being recommended. In addition, potential longer term benefits were also identified, including the ability to federate with other Gen3 based repositories, as well as integration with the Jupyter development environment (Jupyter Notebooks provide a deployable web‐based interface for interactive computing using a range of computing languages: https://jupyter.org/). This decision was sense‐checked by reaching out to Australian colleagues who were also working in the genomics field and using Gen3 (Pope et al., 2022).

The Gen3 technology was adopted, and a bespoke metadata dictionary was built which incorporated existing standards, such as the Genomics Standards Consortium's Minimum Information about any (x) Sequence (MIxS, https://www.gensc.org/pages/projects/mixs‐gsc‐project.html) (Field et al., 2008; Wooley et al., 2009) and several relevant fields not only derived from the Biodiversity Information Standards Organization (TDWG, https://dwc.tdwg.org/) “Darwin Core Standard” (Wieczorek et al., 2012) but also incorporated unique aspects such as the data governance considerations and the adoption of Biocultural Labels and Notices (Anderson & Hudson, 2020); see below for further details. This included several community workshops to further determine what features researchers in Aotearoa New Zealand felt were needed. The current metadata dictionary is available at https://data.agdr.org.nz/DD.

## 3 USING THE AGDR

AGDR has now been in production for approximately eighteen months. At the time of writing, there are 28 Projects hosted, covering 35 experiments/studies across 24 species and two environmental metagenomic studies. The data repository can be browsed, and data access can be requested at https://data.agdr.org.nz/. The exploration page allows for searching and filtering, while the individual project pages provide additional details about each project, including an “apply for access” button.

Although projects and the data they contain are findable using the search and filter functions, access to the data is granted or rejected by individual kaitiaki groups, with differing criteria and internal processes, thus data access is not a uniform process for each data set. Timing to gain approval and access to the data varies from days to weeks depending when the kaitiaki group convene and other scheduling conflicts, as this is all on a voluntary basis. To date, fifteen requests for data access have been received, of which eight have been approved (and researchers have gained access to use the data), four have been rejected, and a further three are under consideration. Where requests were rejected, in all cases it was because requesters did not provide sufficient information on their intended use of the data (despite being prompted for additional information). Other grounds for rejection could include: failure to agree to benefit sharing in a commercial application, desire to use data to assist with a project involving genetic modification, or failure to demonstrate an understanding of Māori cultural principles.

Below we outline the AGDR user‐process with regard to submitting data (“Process for submitting data to the AGDR”, Figure 1), and requesting data access (“Process for requesting access to a data set hosted by AGDR”, Figure 2), referring to the data requirements at submission (Box 3), data access request (Box 4) and some example conditions of data use (Box 5).

We are now seeing publications associated with the data in AGDR across a range of species, including kākāpō (*Strigops habroptilus*) (Guhlin et al., 2023), huia (*Heteralocha acutirostris*) and South Island kōkako (*Callaeas cinereus*) (Dussex et al., 2019), hoki (*Macruronus novaezelandiae*) (Koot et al., 2021), kanakana (*Geotria australis*) (Miller et al., 2022) and metagenomic samples (Tee et al., 2020).

### 3.1 AGDR data submission process

1. The researcher clicks the “Submit data” button on the AGDR website.
2. The researcher is directed to a form that collects basic information about the project in order to determine whether it is appropriate for AGDR and to understand the level of kaitiaki involvement to date. (See Box 4 for a full list of questions).
3. Information is collected and reviewed by a member of the AGDR technical team, who then determine whether to proceed with the submission process. This may involve collecting additional information about the project and/or data set from the research group.
4. If the submission request is approved, the researcher is sent a more detailed request for information, a metadata template spreadsheet, and instructions for the transfer of the data files. A technical team member then contacts the researcher to assist with the data ingest process, and to answer any questions that arise.
5. Once requisite information has been collected, a project instance will be created within the AGDR, and the information will be used to populate the project metadata.
6. If the genomic data have already been generated, a member of the AGDR team will assist with data upload. If data are available for upload, they will be added to the repository. Otherwise the project entry will be created, but not listed on the AGDR website, so that data can be ingested at a later point in time.
7. If Traditional Knowledge or Biocultural Notices and Labels (Anderson & Hudson, 2020) have been created for this data set, links to the Local Contexts Project information are included as project metadata, and the relevant Labels/Notices are added to the AGDR landing page relating to the project.
8. Once project data have been uploaded, researchers are provided the option of having a DOI minted, which allows the data set to be cited.

### 3.2 AGDR data request process

1. The researcher clicks on the “Apply for access” button on the main page for a particular project.
2. The researcher is directed to a form that collects information about their intended use for the data set that they are requesting access to.
3. The request is reviewed for completeness by an AGDR team member. If additional information is required, the requester will be contacted and asked to provide additional details. This includes information relating to the level of iwi/hapū engagement and expected outcomes and any benefits.
4. Once the information is deemed to be sufficient for a decision to be made regarding data access, the request is sent to the individual or group listed as “kaitiaki contact” for this data set. If mana whenua require additional information to assist with the decision‐making process, this is conveyed back to Genomics Aotearoa by the kaitiaki contact, and this information is then requested from the researcher.
5. Once mana whenua have decided whether or not to provide access to the data set, this decision is conveyed back to Genomics Aotearoa, who pass the outcome on to the researcher, along with any feedback from the decision‐making process.
6. If access was approved, the researcher is required to agree to the conditions of data access, and once this is done, a member of the NeSI team will get in touch to provide instructions for data download.
7. The researcher may now download data, which currently are supported through Globus Connect.

## 4 ADDITIONAL CAPABILITIES

### 4.1 Digital object identifiers

As part of, or as a follow on to, the data submission process, the ability to add a Digital Object Identifier (DOI) to a data set is available to researchers using the AGDR. Adding a DOI enables permanent and easy identification outside of AGDR as well as the ability to locate the data set within AGDR. NeSI is a consortium member of Datacite (https://datacite.org/), which enables DOI minting on demand by providing appropriate metadata and links to the data set in the AGDR. To date, 28 DOIs have been generated, and DOI minting is provided as a standard service for all new submissions. Researcher feedback indicates that these are essential for publication in most journals, and are much better than providing a URL to the data, which can subsequently change. As a specific example, the DOI for the “Sequenced genome for rimurapa/southern bull kelp/Durvillaea antarctica” data set is: https://doi.org/10.57748/W5J7‐SM76

### 4.2 Biocultural labels and notices

To facilitate outward‐facing communication of Māori rights over genetic resources to those browsing the repository, the AGDR has collaborated with “Local Contexts” (Anderson & Christen, 2019) to incorporate Biocultural Labels and Notices (Anderson & Hudson, 2020; Liggins, Hudson, & Anderson, 2021). In partnership with Indigenous communities, Local Contexts developed Biocultural (BC) Labels and Notices in response to the Nagoya Protocol and concerns over Indigenous (genomic) Data Sovereignty (Carroll et al., 2021; Hudson et al., 2020). The BC Labels and Notices are easily recognizable icons (similar to Creative Commons Licence icons) that are machine‐readable and easily retained as metadata attached to physical and digital biodiversity resources and data. They were specifically designed to support the inclusion of Indigenous communities and their interests in existing research systems and infrastructures such as AGDR, and are now being used globally in natural history collections, genomic data and metadata repositories, and specific project infrastructures.

The value of incorporating the BC Labels and Notices in AGDR is that they provide kaitiaki and their iwi, hapū and whānau with the option of using an internationally recognized system in support of Indigenous Data Sovereignty. As the international research community becomes more familiar with BC Labels and Notices, it will help users of AGDR to learn about and comprehend the complexities of Māori Data Sovereignty and the interests of Māori in genomic data prior to requesting data access, so that they may be better prepared in their research approach. In some cases, the inclusion of Indigenous species naming may provide additional information about ecological relationships that are not captured by standard taxonomy. The BC Labels and Notices shown in AGDR are generated within the Local Contexts Hub where Indigenous communities have control over the description associated with each instance of BC Label use, communicating their rights and interests associated with a specific project or data set. Labels and Notices are then shared to data sets within AGDR via an API, and are presented on the relevant Project page as icons with a link to the Local Contexts Hub where more complete information can be found.

## 5 DISCUSSION

The creation of the Aotearoa Genomic Data Repository has allowed for genomic data sets to be stored in a manner which is consistent with the principles of Indigenous data sovereignty, as promoted by the United Nations Declaration on the Rights of Indigenous Peoples (United Nations General Assembly, 2007), as well as international groups, such as the Global Indigenous Data Alliance, creators of the CARE Principles of Indigenous Data Governance (Carroll et al., 2021). Although the AGDR is a relatively small undertaking by international standards, it serves as an example of how both data guardianship, and data sharing, can work from an Indigenous peoples' perspective, as well as providing a concrete example (from both cultural and technical perspectives) on how such a repository can be established and operated. With increasing importance being placed on improved mechanisms for Indigenous data sovereignty within the international scientific community, we are hopeful that similar initiatives will soon emerge in this space.

The AGDR is still being developed in response to user feedback and according to research community best‐practices. For instance, having ingested 28 data sets into the repository, the submission process is now being reviewed, with feedback being sought from researchers on their experiences and expectations, as well as the potential to automate some of the currently very manual metadata manipulation. This review will extend to the metadata fields themselves, ensuring they are in keeping with the latest community standards, and to maximize the interoperability of our data sets with those provided by other repositories used by the research community. In addition, follow up with researchers who have had data access requests approved will provide valuable information about their experiences, and will help to facilitate ongoing improvement of this process.

Looking forward, the most immediate challenge for the AGDR is to ensure longevity of operations. Current funding for this initiative is provided via Genomics Aotearoa, a research infrastructure project funded by the NZ Government through the Ministry of Business, Innovation and Employment, through to late 2024. Beyond this, however, additional funding needs to be secured to ensure ongoing operation of the repository, to properly resource kaitiaki in their roles controlling data access and the formation of a kāhui Māori (Māori advisory group), as discussed below.

### 5.1 Costs associated with data access

An additional (and often under‐appreciated) cost associated with the operation of a kaitiaki‐centric data repository is the cost incurred through the ongoing assessment of data access requests. Unlike in academic research settings, where the cost of the time spent by data access committees assessing requests for access to restricted data sets is generally absorbed by researchers' host institutions, discussions among kaitiaki groups could involve broad representation from within communities, with this time essentially being “donated” by participants. As a result, the assessment of such requests has the potential to place an unfair burden on kaitiaki groups, thus disincentivizing interaction with the repository.

Although the funding that is currently available for operating the ADGR covers costs associated with database hosting and maintenance as well as development work and some additional administrative tasks, there is no specific budget available to cover time spent considering access requests. While this may have been appropriate during the construction of a production system (where the focus was squarely on establishing a workable resource), now that the repository has been established, serious consideration should be given to covering *all* of the costs associated with its operation, including those incurred through the kaitiaki assessment process. There are a number of ways in which these costs could be recovered that are currently being explored, including:

1. a user‐pays approach, where data access requests incur a “processing fee” to either fully or partially cover the cost of the assessment process;
2. a “submitter‐pays” approach, where researchers who submit data (or their host institutions) pay a per‐request (or perhaps annual) fee to cover these costs;
3. a remuneration arrangement between research institutions and their Māori research partners, covering (among many other things) the costs associated with acting as kaitiaki;
4. government funding that includes a component to cover kaitiaki assessment costs.

In Aotearoa New Zealand, funding agencies and research institutions are extremely encouraging of research being carried out in partnership with Māori, particularly when processes such as study co‐design are used to ensure that the research produces benefits for the Māori community with which the research is being undertaken. If meaningful research partnerships are to be developed with Māori communities, it is critical for research institutions to establish deep and long‐lasting relationships with those communities, underpinned by agreements that clearly specify benefit sharing and appropriate remuneration for the services that are being provided by both parties.

### 5.2 Establishing an ongoing kāhui Māori

During the establishment phase of AGDR, oversight from a Māori perspective was provided through the Vision Mātauranga coordinator for Genomics Aotearoa, who was embedded within the development team, and was actively involved in the project through the creation of the access and submission protocols. With AGDR now moving into a production setting, additional Māori oversight is required, both with respect to decision‐making around future work to be undertaken, as well as to provide a focus point for the increasing number of kaitiaki groups that are connecting with AGDR as more data sets are submitted. Work is currently underway to establish a kāhui Māori (Māori advisory group), to provide oversight on the governance and operation of the ADGR at a national level, and to help facilitate a co‐design process as the AGDR matures.

### 5.3 International context

The development of the AGDR has arisen in the context of an international shift towards increased acknowledgement of Indigenous rights, which has occurred gradually over many years. International human rights conventions such as the United Nations Declaration on the Rights of Indigenous Peoples (United Nations General Assembly, 2007), provide a rules based ‘minimum standard’ of international obligations for how nation states act towards Indigenous peoples, while international trade agreements are also becoming important instruments for protecting Indigenous rights. For instance, NZ developed the Indigenous Peoples Economic Trade and Cooperation Agreement in 2021, the Comprehensive & Progressive Agreement for Trans‐Pacific Partnership (CPTPP) includes clauses for Māori protection and is now standard for New Zealand Free Trade Agreements, and the Canada–United States–Mexico Agreement (CUSMA) includes similar protections for Indigenous peoples.

In the area of genomics, the Nagoya Protocol on Access to Genetic Resources and the Fair and Equitable Sharing of Benefits Arising from their Utilization to the Convention on Biological Diversity is an international agreement which aims to promote the fair and equitable sharing of benefits that arise from the use of genetic resources, particularly with relevant Indigenous peoples. Although not signatory to the Nagoya Protocol, it is anticipated that Aotearoa New Zealand will adhere to these guidelines, requiring appropriate stewardship of genetic resources and derived Digital Sequence Information, ensuring mutually agreed terms for their use and benefit‐sharing. While global open data repositories are also responding to expectations under the Nagoya Protocol (e.g. INSDC will be requiring ‘Country of Origin’ from the end of 2022, https://www.insdc.org/spatio‐temporal‐annotation‐policy‐18‐11‐2021), these international systems are less agile in brokering the relationship between researchers and Indigenous communities than an in‐country repository such as AGDR. Furthermore, given that several distinct tribes and clans (e.g. iwi, hapū and whānau) can reside within nations, ‘Country of Origin’ may fail to identify the specific Indigenous Peoples from whom consent should be received. In this way, we expect that AGDR, which attributes data to relevant kaitiaki and their iwi, hapū and whānau, will become a necessary national resource, and a useful exemplar for other nations.

### 5.4 Molecular ecology applications

With the consent of kaitiaki responsible for data held within AGDR, the benefits of a national repository of genomic data can also extend into conservation and management. For instance, monitoring genetic diversity within species is central to global biodiversity strategies such as Aichi Target 13 (i.e. “minimize genetic erosion” and “safeguard genetic diversity”) and Sustainable Development Goal 2.5 as well as the national biodiversity strategy ‘Te Mana o te Taiao’ to ensure populations are genetically diverse and buffered from impacts arising from a loss of genetic diversity (New Zealand Department of Conservation, 2020). However, few nations, including Aotearoa New Zealand, are quantitatively monitoring and reporting on genetic diversity (Hoban et al., 2021), and existing genomic data have not been routinely incorporated into monitoring, conservation management or decision‐making (Laikre et al., 2010, 2020). This lack of monitoring has in part been due to inadequate stewardship of genomic data and associated metadata that enables data to be temporally and spatially contextualized, and interoperable with other data sets (Toczydlowski et al., 2021). The uptake and routine use of genetic diversity in biodiversity monitoring requires the collation of population genomic data at a national scale, along with metadata that is relevant to domestic priorities (Liggins, Noble, & Network, 2021). In time, the AGDR could become a central repository for these genomic data, and by requiring specific metadata, it will be fit to support future molecular ecological research and contributions (Liggins et al., 2022) including national genetic biodiversity monitoring.

### 5.5 Closing thoughts

The establishment of the AGDR fills an important gap for the genomic research community of Aotearoa New Zealand, providing a mechanism for in‐nation data storage and management, implemented in a way which respects and enables Māori data sovereignty. It also provides a focal point for kaitiaki groups involved in genomic research, thus facilitating conversations about data management, research partnership and benefit sharing between researchers, their host institutions and the Indigenous communities with whom they have research partnerships. The future success of science initiatives lies in bringing communities along in the journey of knowledge creation. AGDR provides infrastructure for this journey in relation to Indigenous People.

## BENEFIT‐SHARING STATEMENT

Benefits generated: this project has generated a resource that can help researchers, their host institutions, and the communities they work with fulfil the management, sharing and kaitiaki responsibilities associated with genomic data sets generated from taonga species. The initiative has been undertaken by two major providers of national research infrastructure in Aotearoa New Zealand (Genomics Aotearoa and NeSI), and through engagement with the scientific community, has helped to increase the awareness of Indigenous data sovereignty in the context of genomic research.

## AUTHOR CONTRIBUTIONS

BTA, MAB, LL, CR, JH, EOP and RB designed the research. Technical development and implementation were carried out by CR, JH and EOP, and development of cultural protocols was carried out by BTA. Additional cultural oversight was provided by TG. All authors contributed to the writing and editing of the manuscript.

## CONFLICT OF INTEREST STATEMENT

The authors declare that there are no conflicts of interest.

## Data Availability

No data were generated for this article. Access to all AGDR‐hosted data sets mentioned in this article may be requested at https://data.agdr.org.nz.
